# Safety and clinical activity of the Notch inhibitor, crenigacestat (LY3039478), in an open-label phase I trial expansion cohort of advanced or metastatic adenoid cystic carcinoma

**DOI:** 10.1007/s10637-019-00739-x

**Published:** 2019-04-06

**Authors:** C. Even, U. Lassen, J. Merchan, C. Le Tourneau, J-C Soria, C. Ferte, F. Ricci, J. T. Diener, E. Yuen, C. Smith, G. J. Oakley, K. A. Benhadji, Christophe Massard

**Affiliations:** 1 Drug Development Department (DITEP), Institut Gustave Roussy Cancer Campus, 114 rue Edouard Vaillant, 94800 Villejuif Cedex, France; 2 The Finsen Centre, Rigshospitalet, Copenhagen, Denmark; 3 University of Miami Miller School of Medicine, Miami, FL USA; 4 Department of Drug Development and Innovation (D3i), Institut Curie, Paris, France; 5 INSERM U900 Research Unit, Saint-Cloud, France; 6 Paris-Saclay University, Paris, France; 7 University Paris-Sud, Orsay, France; 8 Eli Lilly and Company, Indianapolis, IN USA

**Keywords:** Adenoid cystic carcinoma, Notch pathway, LY3039478, Crenigacestat, Phase 1

## Abstract

*Background* Deregulated Notch signaling is implicated in multiple cancers. The phase I trial (I6F-MC-JJCA) investigated the safety and anti-tumor activity of crenigacestat (LY3039478), a selective oral Notch inhibitor, in an expansion cohort of patients with adenoid cystic carcinoma (ACC) who received the dose-escalation-recommended phase 2 dose (RP2D), established previously (Massard C, et al., *Annals Oncol* 2018, 29:1911–17). *Methods* Patients with advanced or metastatic cancer, measurable disease, ECOG-PS ≤1, and baseline tumor tissue were enrolled. Primary objectives were to identify a safe RP2D, confirm this dose in expansion cohorts, and document anti-tumor activity. Secondary objectives included safety and progression-free survival (PFS). The ACC expansion cohort received the RP2D regimen of 50 mg crenigacestat thrice per week in a 28-day cycle until disease progression or other discontinuation criteria were met. *Results* Twenty-two patients with ACC were enrolled in the expansion cohort (median age of 60 years). Median treatment duration was 3 cycles with 6 patients remaining on treatment. There were no objective responses; 1 (5%) patient had an unconfirmed partial response. Disease control rate was 73% and 4 patients had stable disease ≥6 months. Median PFS was 5.3 months (95%CI: 2.4-NE)) for the 22 patients; and 7.7 months (95%CI: 4.0-NR) and 2.4 months (95%CI: 1.1-NE) in the subgroup of patients in second-line (*n* = 7) or ≥ third-line (*n* = 9), respectively. Frequent treatment-related-adverse events (all grades) included diarrhea, fatigue, vomiting, decreased appetite, dry mouth, and dry skin. There were no new safety signals. *Conclusion* The crenigacestat RP2D regimen induced manageable toxicity and limited clinical activity, without confirmed responses, in heavily pretreated patients with ACC.

## Introduction

Adenoid cystic carcinoma (ACC) is a rare form of carcinoma characterized by slow growth, frequent recurrences, and a high incidence of metastasis. Surgical resection followed by radiation is the most common treatment protocol; to date, no chemotherapy or drug combination is effective [1, 2].

Notch signaling is an evolutionarily conserved pathway that plays an integral role in development and tissue homeostasis [3, 4]. The oncogenic functions of Notch signaling include the inhibition of apoptosis and the promotion of cell proliferation [5–13]. Recent studies suggest that Notch1 plays a key role in the cell growth and metastasis of ACC, and patients with Notch1 mutations appear to have a more aggressive disease with a distinct pattern of metastasis and worse prognosis [14–16]. Thus, targeting Notch1 represents a potential therapeutic strategy.

Crenigacestat is a potent small molecule inhibitor of Notch cleavage that prevents release of the notch intracellular domain (NICD) by inhibiting proteolytic activity of γ-secretase complex and thereby decreasing Notch signaling and its downstream biologic effects. Crenigacestat inhibits tumor growth in patient-derived tumors representing colon, lung, ovarian, gastric, breast cancer, non-small cell lung cancer, and glioblastoma [17].

Study I6F-MC-JJCA was designed and conducted as a phase 1, nonrandomized, open-label, multicenter trial that evaluated the safety and antitumor activity of crenigacestat in patients with advanced or metastatic cancers. In the dose escalation part of this trial, crenigacestat was well tolerated at doses engaging the Notch receptor in heavily pre-treated patients, including patients with ACC [18]. Crenigacestat exposure was dose proportional. Preliminary pharmacodynamic analyses showed that at a starting dose of 45 mg administered 3 times per week (TIW) in a 28-day cycle, crenigacestat inhibits Notch-regulated gene expression by approximately 50%. Compared with the 75 mg dose, the 50 mg dose was associated with milder to moderate severity of adverse events, commonly diarrhea and vomiting, and a few grade 3 or 4 events. Clinical benefit was observed in 11% of the patients. Thus, given the relative safety/tolerability and potential for clinical benefit/anti-tumor activity, the recommended phase 2 dose (RP2D) of crenigacestat monotherapy was determined as 50 mg administered 3 TIW in a 28-day cycle [18].

Here, we report the safety, tolerability and anti-tumor activity of crenigacestat monotherapy in the confirmatory, expansion cohort of patients with ACC enrolled in I6F-MC-JJCA who were treated with the RP2D.

## Methods

### Study design and treatment

I6F-MC-JJCA was a phase 1 multi-center, nonrandomized, open-label, first-in-man trial of oral crenigacestat (LY3039478), in patients with advanced or metastatic cancer (ClinicalTrials.gov identifier: NCT01695005). Primary objectives were to evaluate the safety of crenigacestat in a dose-escalation phase and determine a RP2D, to confirm the RP2D of crenigacestat in the expansion cohort, and to document anti-tumor activity. Secondary objectives included characterizing the safety and toxicity profile of crenigacestat and assessing the duration of response and progression-free survival (PFS). Exploratory objectives examined potential predictive biomarkers, pharmacodynamic effects of crenigacestat, and utility of positron emission tomography (PET) scan to assess the treatment effect of crenigacestat.

In the confirmatory, expansion cohort, patients received the RP2D of 50 mg crenigacestat TIW in 28-day cycles until symptomatic or confirmed progressive disease, unacceptable toxicity, or other study drug discontinuation criteria were met.

This study was conducted in compliance with the Declaration of Helsinki, Council for International Organizations of Medical Sciences International Ethical Guidelines, International Conference on Harmonization Guidelines for Good Clinical Practice, and applicable local regulations. The ethics committees of all participating centers approved the protocol, and all patients provided written informed consent before study entry.

### Patients

Eligible patients were ≥ 18 years of age and had histological evidence of advanced or metastatic ACC. All patients had measurable disease or reliable biomarker measure. Patients had an Eastern Cooperative Oncology Group performance status (ECOG-PS) score of ≤1, adequate organ and hematologic functions. Patients were excluded from the study if they had received treatment with a drug that had not received regulatory approval for any indication within 14 or 21 days of the initial dose of study drug and had any serious pre-existing medical conditions. Patients were also excluded if they had any central nervous system malignancy, acute leukemia, or if they had undergone any autologous or allogeneic stem-cell transplantation.

### Study assessments

#### Efficacy assessments

Tumor responses were measured using the appropriate guidelines (RECIST 1.1) [19]. Objective response rate (ORR) was the proportion of patients who achieved a complete response (CR), partial response (PR), or stable disease (SD) out of all the patients who received at least 1 dose of study drug. Best response was determined from a sequence of responses assessed. Minimum change in tumor size from baseline was presented in a waterfall plot for patients with measureable lesion.

Use of PET scan to assess treatment effect of crenigacestat was mandatory for the ACC expansion cohort. Partial metabolic response by PET scan was defined as a minimum of 15% in tumor [18F]-FDG SUV after 1 cycle of therapy and greater than 25% after more than 1 treatment cycle, according to PET response criteria of the European Organization for Research and Treatment of Cancer [20].

#### Safety assessments

All adverse events (AEs) were coded according to the Medical Dictionary for Regulatory Activities, version 19.0 and graded by National Cancer Institute’s (NCI) Common Terminology Criteria for Adverse Events (CTCAE) 4.0. Dose-limiting toxicity (DLT) was defined as an AE during Cycle 1 that is related to crenigacestat and fulfills any one of the following criteria using the NCI CTCAE v 4.0: ≥3 CTCAE Grade 3 nonhematological toxicity (exceptions made for nausea, vomiting, or constipation that lasts <72 h and can be controlled with treatment; transient Grade 3 elevations of ALT and/or AST), CTCAE Grade 4 hematological toxicity of >5 days duration, any febrile neutropenia, Grade 3 thrombocytopenia with bleeding or Grade 4 thrombocytopenia, and other significant toxicity deemed to be dose limiting by investigator.

#### Pharmacokinetic assessments

Crenigacestat PK parameter estimates for patients were summarized and compared to PK parameters from the dose-escalation phase of the trial. Plasma samples were collected up to 4 h after the first dose for PK evaluation.

#### Exploratory biomarker assessments

Patients in the study submitted representative pre-treatment archival diagnostic biopsies as formalin-fixed, paraffin-embedded (FFPE) tissue, with some patients submitting pre- and post-treatment biopsies collected in formalin or PAXgene® Tissue Fix. Specimens were sectioned at 4–5 μm (if submitted as blocks) onto positively charged slides and baked at 60 °C for at least 15 min or until dry. De-paraffinization and antigen retrieval were accomplished using EnVision™ FLEX Target Retrieval Solution, High pH (K8000; Dako, Carpinteria, CA, USA) in a Dako PT Link unit reaching 97 °C for 20 min. The retrieved NICD was detected using the Dako EnVision™ FLEX+ Rabbit visualization system (K8009) on the Dako Autostainer Link 48 automated slide stainer with proprietary 1.5 μg/mL N1ICD, 2.0 μg/mL N2ICD, or 2.0 μg/mL N3ICD antibody developed for Eli Lilly and Company. Results were interpreted and scored by a board-certified pathologist (GJO). Specimens were scrutinized for the level of endogenous background signal by examining additional sections using an isotype control. Control tissues were processed in parallel with tissues exposed to primary antibody. Immunohistochemistry (IHC) results were scored by a qualitative method based on an assessment of the immunoreactivity observed in the specimen using a scale of 0 to 3+ translating to no (0), weak (1+), moderate (2+), or intense (3+) staining, respectively. A cut-off of ≥10% tumor cells with specific nuclear staining with ≥1+ immunoreactivity was used to determine positive immunoreactivity.

### Statistical analyses

Data from all patients who received at least 1 dose of crenigacestat treatment were included in summaries of safety and efficacy. Analyses of safety and efficacy were based on October 2016 data transfer.

Change in tumor size was assessed in each patient with measurable disease using radiographic imaging. The minimum change in tumor size was summarized for all patients with a pre- and post-treatment assessment using a waterfall plot. For patients evaluated by PET scan, the minimum change in SUV_max_ was summarized using a waterfall plot. ORR and PET metabolic response rates were summarized descriptively. Descriptive analyses of PFS and OS were conducted using the Kaplan-Meier method. Plasma and urine concentrations were measured using validated LC/MS/MC methods.

## Results

### Patient characteristics

Twenty-two patients with ACC were treated with crenigacestat monotherapy (Table 1) of which 13 (59%) were male and 9 (41%) were female. The majority (82%) of patients had ECOG-PS 1. Median age was 60 years (range 41–82). 20 (91%) patients had prior radiotherapy and 9 (41%) patients received two or more prior systemic treatments. All patients had metastatic disease and 14 (64%) patients were positive for Notch 1.

**Table 1:** Patient and disease characteristics

Characteristics	N = 22, n (%)
Gender	
Male	13 (59%)
Female	9 (41%)
Age, years, median (range)	60 (41–82)
Race	
White	19 (86%)
African American	1 (5%)
Missing	2 (9%)
ECOG	
0	4 (18%)
1	18 (82%)
Prior therapy	
Surgery	14 (64%)
Radiotherapy	20 (91%)
Prior systemic treatments	
0	6 (27%)
1	7 (32%)
≥ 2	9 (41%)
Metastatic Disease	22 (100%)
Sites of metastases	
Lung	20 (91%)
Bone	8 (36%)
Liver	5 (23%)
Lymph node	5 (23%)
Notch ICD IHC	
Positive	14 (64%)
Negative	8 (36%)

*ECOG*, Eastern Cooperative Oncology Group; *NICD*, Notch-1 intracellular domain

### Efficacy

**Fig. 1:**
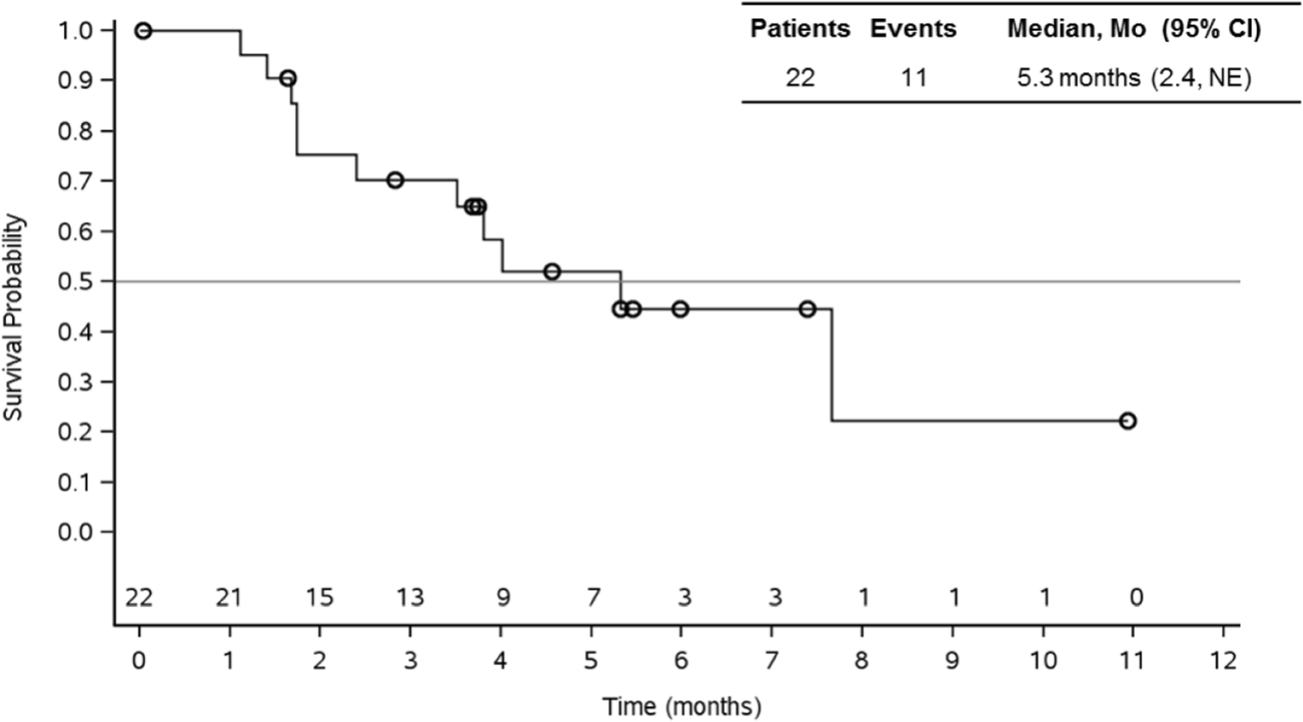
Progression-free survival

**Fig. 2:**
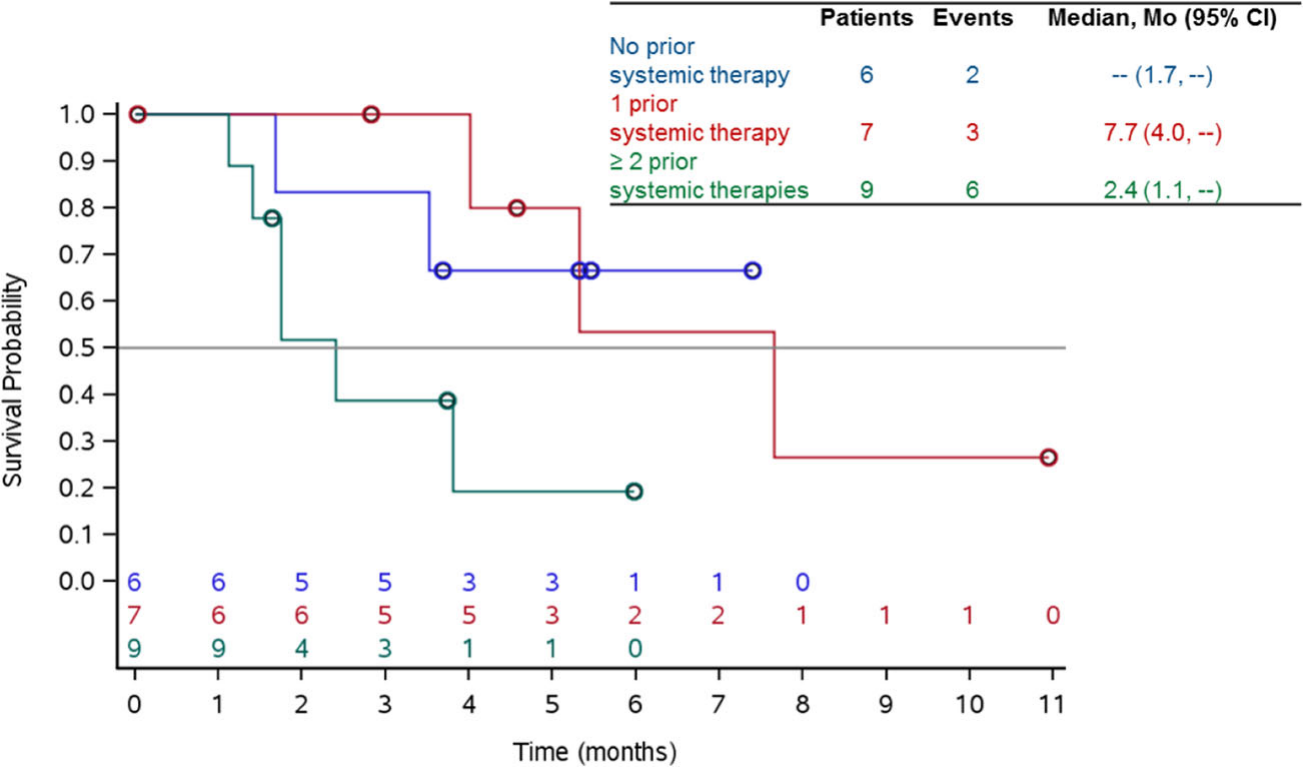
Progression-free survival by lines of prior systemic therapies

Median treatment duration was 3 cycles (range 1–10) with 6 patients remaining on treatment. 1 (5%) patient had an unconfirmed partial response. A total of 15 (68%) patients had stable disease of which 4 patients had stable disease for ≥6 months. 5 (23%) patients had progressive disease. Disease control rate (DCR) was 73% (16 out of 22 patients) (Table 2). In the overall group (*n* = 22), median PFS was 5.3 months (95% CI: 2.4-NE) **(**Fig. 1**).** Median PFS was 7.7 (95% CI: 4.0-NE) for patients in second line therapy (*n* = 7), while it was 2.4 (95% CI: 1.1- NE) for patients in third line or more (*n* = 9). In patients without prior systemic therapy (*n* = 6), median PFS could not be estimated since 4 of those patients were censored **(**Fig. 2**).**

**Table 2:** Summary of best overall response

	N = 22 n (%)
Partial response (unconfirmed)	1 (5%)
Stable disease (SD)	15 (68%)^a^
Progressive disease (PD)	5 (23%)
Not evaluable	1
Disease control rate (CR + PR + SD)	16 (73%)

^a^4 patients had stable disease ≥6 months

### Patient disposition

**Fig. 3:**
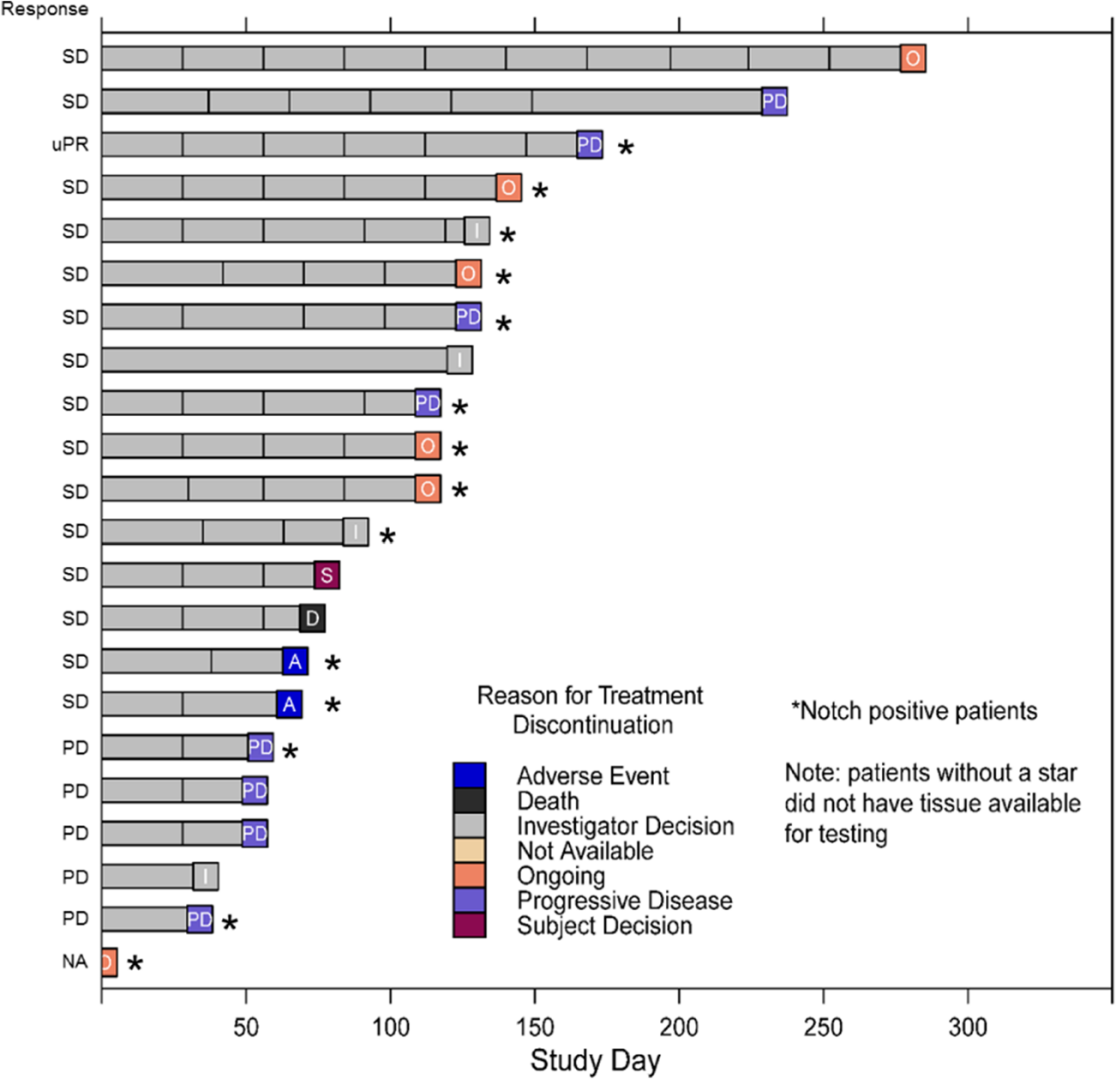
Tumor response over time. uPR = unconfirmed partial response, SD = stable disease, PD = progressive disease, NA = not available

At the time of data cut off, 6 out of 22 patients were still on treatment and 16 patients had discontinued the treatment (Fig. 3). A total of 8 patients discontinued due to progressive disease while 4 patients discontinued due to investigator’s decision. Other reasons for discontinuation were AEs (*n* = 2; 1 patient discontinued due to emergence of a squamous cell skin carcinoma), withdrawal by subject (*n* = 1), or death (n = 1; sudden death unrelated to study drug).

### Safety and tolerability

The majority of AEs in all the patients receiving crenigacestat were mild to moderate in severity, with a few patients experiencing Grade 3/4 events. 3 (13.6%) patients experienced Grade 3/4 diarrhea. Hypophosphatemia, colitis, nausea, fatigue, increased ALT, increased AST, increased blood creatinine, and dehydration were the most common Grade 3/4 AEs, which occurred in at least 1 patient. There were 2 (9.1%) patients who reported Grade 3/4 squamous cell carcinoma of skin. Most frequent treatment-emergent drug related adverse events (all grades) occurring in ≥10% of patients were diarrhea (*n* = 12; 55%), fatigue (*n* = 10; 45%), vomiting (*n* = 8; 36%), dry mouth (*n* = 6; 27%), decreased appetite (n = 6; 27%), dry skin (*n* = 5; 23%), hypophosphatemia (*n* = 4; 18%), stomatitis (n = 4; 18%), nausea (n = 4; 18%), dysgeusia (n = 4; 18%), hair color changes (n = 4; 18%), pyrexia (*n* = 3; 14%), increased ALT (n = 3; 14%), decreased weight (n = 3; 14%), alopecia (n = 3; 14%), and rashes (n = 3; 14%) (Table 3).

**Table 3:** Most frequent related adverse events (≥10% of patients)

Adverse events	Investigator-determined Maximum CTC Grade, n (%)				N = 22 n (%)
	Grade 1	Grade 2	Grade 3	Grade 4	
Diarrhea	3 (14)	6 (27)	3 (14)	–	12 (55)
Fatigue	6 (27)	3 (14)	1 (5)	–	10 (45)
Vomiting	4 (18)	4 (18)	–	–	8 (36)
Dry mouth	6 (27)	–	–	–	6 (27)
Decreased appetite	3 (14)	3 (14)	–	–	6 (27)
Dry skin	4 (18)	1 (5)	–	–	5 (23)
Hypophosphatemia	2 (9)	2 (9)	–	–	4 (18)
Dysgeusia	4 (18)	–	–	–	4 (18)
Stomatitis	3 (14)	1 (5)	–	–	4 (18)
Nausea	2 (9)	2 (9)	–	–	4 (18)
Hair color changes	3 (14)	1 (5)	–	–	4 (18)
Pyrexia	3 (14)	–	–	–	3 (14)
ALT increased	1 (5)	1 (5)	1(5)	–	3 (14)
Weight decreased	1 (5)	2 (9)	–	–	3 (14)
Alopecia	3 (14)	–	–	–	3 (14)
Rash	3 (14)	–	–	–	3 (14)

*ALT*, Alanine aminotransferase

#### Pharmacokinetics

PK was assessed in 17 patients, with maximum plasma concentrations (C_max_) occurring approximately 2 h post-dose following 50 mg TIW oral doses of crenigacestat. The geometric mean C_max_ was approximately 418 ng/mL and area under the plasma concentration time curve from time 0 to 4 h [AUC_(0–4)_] was approximately 1070 ng*h/mL. These PK parameters appeared similar to those calculated from patients who underwent intensive PK sampling in the dose-escalation portion of I6F-MC-JJCA and the exposures achieved were similar to other patients with advanced cancer.

#### Exploratory biomarker and pharmacodynamics analyses

**Fig. 4:**
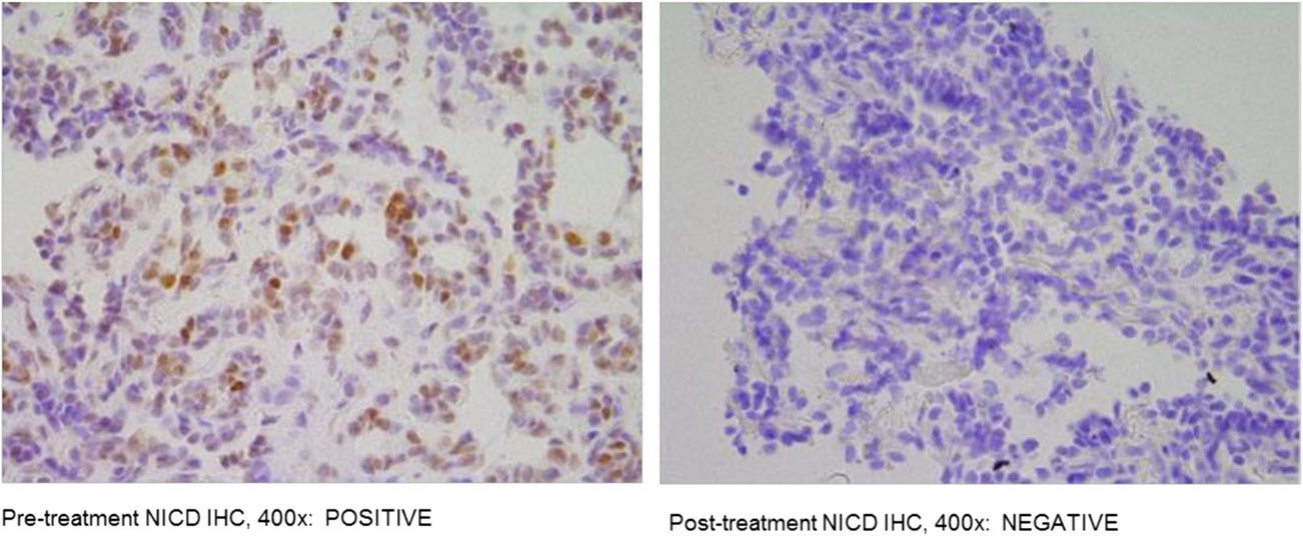
Pre- and post-treatment tumor biopsies (Notch IHC). Duration of stable disease 3.5 months. *This was accessed only in one patient. So, further evaluation in future is needed

Pre- and post-treatment tumor biopsies were assessed for alterations in the Notch pathway. Notch activation via detection of the active NICD fragment by IHC was found in a number of pre-treatment samples. 14 out of 22 (64%) patients with evaluable samples were positive for Notch 1 IHC. Two patients had pre- and post-treatment biopsies, which were evaluable. In the post-treatment samples, 1 patient was negative for Notch staining and the other was positive. Duration of stable disease in these patients was 3.5 months and 6 months, respectively. Pre- and post-treatment tumor biopsies (histology and CD8IHC) are illustrated in Fig. 4.

**Fig. 5:**
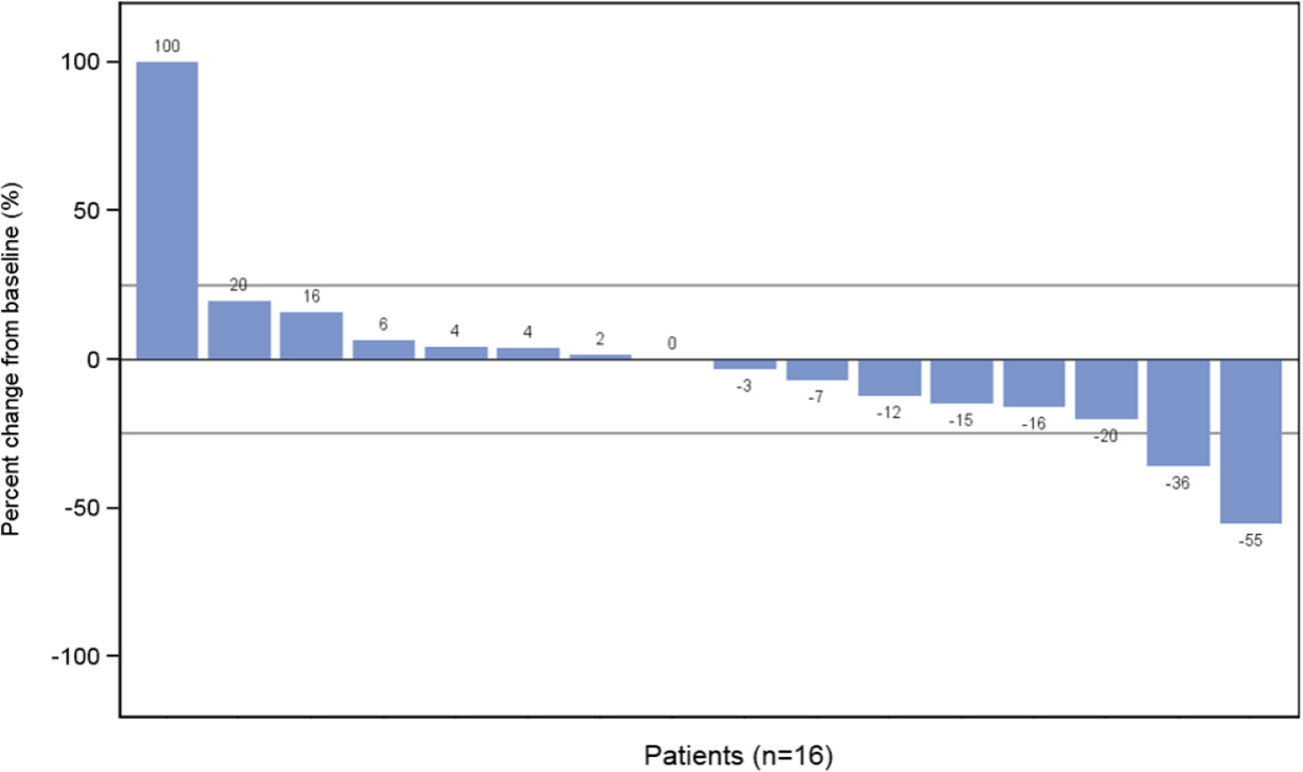
Minimum Change in SUV_max_ by PET Assessment

In preliminary analysis, 16 patients were assessed for minimum change in SUV_max_ by PET, of which 2 (13%) had unconfirmed partial metabolic response (Fig. 5).

## Discussion

This report describes the phase I safety, tolerability, and clinical activity of crenigacestat, a highly potent and selective Notch inhibitor of ACC. We have established that crenigacestat can be administered safely on a TIW schedule at a dose of 50 mg in patients with ACC. Grade 3/4 toxicities were low. Similar to the patients in the previously reported dose escalation and other dose expansion cohorts reported for this trial [18, 21], gastrointestinal toxicity (diarrhea and nausea) was the major toxicity observed in these patients. These events were consistent with the previously reported clinical safety profile for Notch pathway inhibitors.

There were no objective partial or complete responses. However, a majority of the patients (58%) with ACC achieved stable disease and 4 patients among them had stable disease for more than 6 months despite the patient population being heterogeneous and heavily pretreated, with more than 40% receiving crenigacestat monotherapy as second line or greater. Yet, ACC is unique in its unpredictable, yet slow-growing, nature [22]. Despite this and the fact that these patients were heavily pre-treated, overall, disease control rate was 73% (16/22). Two out of 16 (13%) patients demonstrated unconfirmed partial metabolic response evidenced by reduction in PET SUV_max_. The geometric mean C_max_ and area under plasma concentration time curve appeared similar to those calculated from patients who underwent intensive PK sampling in the dose-escalation phase.

A change in Notch expression pattern was observed in patients who underwent pre- and post-treatment biopsies, which may be consistent with pharmacologic activity; however, samples were submitted in several types of fixative, which could impact the sensitivity and specificity for activation of Notch by IHC. The high level of Notch activation in our patient cohort’s pre-treatment samples is consistent with recent studies of Notch activation prevalence in ACC [23, 24], Differences seen, i.e., the slightly lower percentage observed, however, may be due to fixation techniques and IHC antibody clones.

In summary, the results of the ACC expansion cohort in this phase 1 trial demonstrate that crenigacestat has a manageable safety profile and a clinical pharmacodynamic effect on Notch-targeted genes. However, crenigacestat clinical activity was limited at the recommended dose with no confirmed objective response. Trials are ongoing to explore crenigacestat in combination with targeted agents and chemotherapy. Overall, this work supports the rationale for targeting Notch signalling and further implicates Notch signalling in tumor physiology.
